# Corticosteroids and ARDS: A review of treatment and prevention evidence

**DOI:** 10.4103/0970-2113.80324

**Published:** 2011

**Authors:** G.C. Khilnani, Vijay Hadda

**Affiliations:** *Department of Medicine, Pulmonary and Critical Care Division, All India Institute of Medical Sciences, New Delhi, India*

**Keywords:** Acute respiratory distress syndrome, adult respiratory distress syndrome, corticosteroids, methylprednisolone

## Abstract

To systematically review the role of corticosteroids in prevention of acute respiratory distress syndrome (ARDS) in high-risk patients, and in treatment of established ARDS. Primary articles were identified by English-language Pubmed/MEDLINE, Cochrane central register of controlled trials, and Cochrane systemic review database search (1960–June 2009) using the MeSH headings: ARDS, adult respiratory distress syndrome, ARDS, corticosteroids, and methylprednisolone (MP). The identified studies were reviewed and information regarding role of corticosteroids in prevention and treatment of ARDS was evaluated. Nine trials have evaluated the role of corticosteroid drugs in management of ARDS at various stages. Of the 9, 4 trials evaluated role of corticosteroids in prevention of ARDS, while other 5 trials were focused on treatment after variable periods of onset of ARDS. Trials with preventive corticosteroids, mostly using high doses of MP, showed negative results with patients in treatment arm, showing higher mortality and rate of ARDS development. While trials of corticosteroids in early ARDS showed variable results, somewhat, favoring use of these agents to reduce associated morbidities. In late stage of ARDS, these drugs have no benefits and are associated with adverse outcome. Use of corticosteroids in patients with early ARDS showed equivocal results in decreasing mortality; however, there is evidence that these drugs reduce organ dysfunction score, lung injury score, ventilator requirement, and intensive care unit stay. However, most of these trials are small, having a significant heterogeneity regarding study design, etiology of ARDS, and dosage of corticosteroids. Further research involving large-scale trials on relatively homogeneous cohort is necessary to establish the role of corticosteroids for this condition.

## INTRODUCTION

Acute respiratory distress syndrome (ARDS) is an acute hypoxemic state caused by the sudden development of diffuse injury to the terminal respiratory units with exudative pulmonary edema and is associated with very high mortality. The management of ARDS has evolved over decades and there is consistent increase in survival among these patients over the last few years.[1] Improved critical care, including medical technology, better-trained staff, and better infection control, with newer and potent antibiotics has contributed to improved outcome in these patients. Currently, most of the patients survive the initial insult that precipitates ARDS. However, no pharmacological protocol is effective in modifying the course of this condition which still has mortality rate of 30 to 50% and high morbidity in intensive care units (ICU).[2,3]

It is well understood that inflammation is the key factor in the pathophysiology of ARDS, irrespective of etiology. Furthermore, in the inflammatory cascade, insufficient glucocorticoid receptor-mediated inhibition of proinflammatory NF-_k_B is thought to be central to the pathogenesis of ARDS.[2] This inflammatory injury to the lungs evolves through various stages which include early stage or exudative phase followed by proliferative and late stage or fibrotic phase.[4,5] Early or exudative phase is characterized by intense inflammatory response with alveolar macrophages secreting cytokines, interleukin-1, 6, 8, and 10, and tumor necrosis factor α, which act locally to stimulate chemotaxis and activate neutrophils. Neutrophils release oxidants, proteases, leukotrienes, and other proinflammatory molecules, such as platelet-activating factor. The final result is sloughing of both the bronchial and alveolar epithelial cells, with the formation of protein-rich hyaline membranes on the denuded basement membrane. The influx of protein-rich edema fluid into the alveolus has led to the inactivation of surfactant. Following this acute phase of the ARDS, some patients have an uncomplicated course with rapid resolution of the disorder. However, persistent ARDS is characterized by ongoing inflammation,[6,7] parenchymal-cell proliferation,[4] and disordered deposition of collagen.[4,8–10] Late phase of ARDS is characterized histologically by mesenchymal cell proliferation and neovascularization of alveolar space and these finding are associated with increased risk of death.

Most of the cases of ARDS are reversible; therefore, reversing the adverse physiological state and tiding over the crisis is expected to improve the outcome. Ventilatory strategies for ARDS have evolved over the period and use of low tidal volume ventilation and appropriate use of positive end expiratory pressure had a favorable impact on outcome in these patients.[11] However, there is continuous search for an effective pharmacological agent which would improve the outcome of patients suffering with this syndrome. Pharmacotherapeutic trials to modify the course of this condition with various agents such as surfactant,[12] inhaled nitric oxide,[13] antifungal drugs,[14] and N-acetyl cysteine[15,16] at various stages did not show encouraging results. Some studies with corticosteroids have shown favorable results, while others have shown no benefits. However, these agents have several adverse effects; therefore, should only be used if benefit outweighs the risk. This review summarizes the available evidence of utility of corticosteroids in the management of ARDS.

## CORTICOSTEROIDS AS POTENTIAL THERAPEUTIC AGENT FOR ARDS

Corticosteroids are a group of natural and synthetic analogues of hormones secreted by the hypothalamic-pituitary-adrenocortical axis. These drugs are potent anti-inflammatory, antifibrotic, and immunomodulator agents which exert inhibitory effects at several stages of the inflammatory cascade.[17] These drugs exhibit their anti-inflammatory activity through the following three independent mechanisms: the induction and activation of annexin-I, induction of mitogen-activated protein kinase phosphatase-1, and repression of transcription of cyclooxygenase-2, resulting in blockade of transcription of various cytokines, chemokines, cell adhesion molecules, and complement factors responsible for development of ARDS.[18–20]

Considering the fact that inflammation is central to the pathogenesis of ARDS, corticosteroids would be a logical choice for the management of ARDS.[17] Following the above concept, researchers have tried corticosteroids at various stages of ARDS.[21–25] These agents also have been tried for prevention of this potentially fatal complication in high-risk patients.[26–29]

### Data sources and selection

Primary articles were identified by English-language Pubmed/MEDLINE, Cochrane central register of controlled trials, and Cochrane systemic review database (1960–June 2009) search using the MeSH headings: Acute respiratory distress syndrome, adult respiratory distress syndrome, ARDS, corticosteroids, and methylprednisolone (MP). All studies published in the English language that evaluated the role of corticosteroids in management of ARDS in adults and adolescents were analyzed and pertinent articles were selected for this review.

## CORTICOSTEROIDS FOR PREVENTION OF ARDS

ARDS develops after a variety of insults, including sepsis, multiple trauma, pneumonia, aspiration of gastric contents, and severe burns. Its pathogenesis includes immune-mediated injury leading to loss of the alveolar-capillary barrier, injury to the alveolar epithelium, an influx of neutrophils and macrophages, and fibrin. These changes develop over hours to a few days after the initiating event. Therefore, timely modulation of inflammation using corticosteroids in these high-risk patients would be a rational approach for prevention of ARDS and its consequences.

There are only a few trials on the role of corticosteroids as immunomodulators to prevent development of ARDS in high-risk patients, such as patients with severe sepsis.[26–29] However, results were not encouraging. Weigelt *et al*., in a double blind trial in patients with respiratory failure admitted to surgical ICU, have shown that MP given as 30 mg/kg IV every 6 hours for 48 hours did not prevent ARDS in high-risk patients.[26] Contrary to expectations, there were more patients in corticosteroid group (64%) as compared with placebo (33%) who developed ARDS. This study also demonstrated that corticosteroids use was associated with increased incidence of infectious complications (77% *vs* 43%).[26] Similar results were shown by Bone *et al*. in a larger cohort.[28] In this study, 382 patients with sepsis were randomized to receive either MP (30 mg/kg every 6 hours for 4 doses) or placebo. This study showed that there was a trend toward increased incidence of ARDS in MP group (32%) as compared with placebo (25%), though results were not statistically significant. However, mortality at day-14 was significantly higher (52% *vs* 22%; *P* = 0.005) in patients having received MP as compared with placebo. Other studies [Table 1] also showed that prophylactic short course high-dose corticosteroids were associated with increased chances of developing ARDS and/or mortality.[27,29]

**Table 1:** Trials of corticosteroids for prevention of ARDS

Author, Year^[Ref]^	Drug/dosage	No. patients developing ARDS/total no patients (%)		OR (95% CI)	*P* value
		Corticosteroid group	Placebo group		
Weigelt *et al*. 1985[26]	Methylprednisolone 30 mg/kg 6 hourly for 48 hours	25/39 (64.1)	14/42 (33.3)	2.36 (1.14-6.28)	0.008
Schein *et al*. 1987[27]	Methylprednisolone 30 mg/kg or Dexamethasone 6 mg/kg single dose	7/29 (24.1)	2/13 (15.3)	1.48 (0.48-4.44)	Not mentioned
Bone *et al*. 1987[28]	Methylprednisolone 30 mg/kg 6 hourly for 24 hours	50/152 (32.9)	38/152 (25)	1.48 (0.93-2.34)	0.10
Luce *et al*. 1988[29]	Methylprednisolone 30 mg/kg 6 hourly for 24 hours	13/38 (34.2)	14/37 (37.8)	1.55 (0.44-2.32)	Not significant

ARDS: Acute respiratory distress syndrome

It is evident from these clinical trials that corticosteroids have no role in prevention of ARDS in high-risk patients. On the contrary, it leads to higher rates of ARDS, increased infectious complications, and mortality. Therefore, we strongly argue against prophylactic use of corticosteroids in seriously ill patients who are prone to develop ARDS.

It is important to note that in all these studies, MP was used for a maximum duration of 48 hours and its circulating half life in ARDS patients varies from 3.8 to 7.2 hours; therefore, the effect of this drug will diminish after 24 to 36 hours at the most.[30–32] Animal studies have shown that in acute lung injury, prolonged corticosteroids were shown to be effective in reducing edema and collagen deposition in lung, while premature withdrawal rapidly negated these positive effects.[33,34] This seems a plausible explanation for the negative results observed in these studies with short course of high-dose MP. Furthermore, it may be noticed that all these studies have used high doses of MP for prevention of ARDS. However, we have seen that the low-dose corticosteroids are useful in other critical illnesses such as septic shock.[35,36] Therefore, one may argue that in addition to short duration, high doses might have been responsible for the negative results.

## CORTICOSTEROIDS FOR THE TREATMENT OF ARDS

Initially, case reports showed that prolonged use of corticosteroids improves lung function in patients with unresolving ARDS.[37,38] Also, trials had shown that improvement in lung functions were associated with survival in these patients.[22,39] Based on these results, researchers tried prolonged course of corticosteroids in patients with established ARDS at various stages, that is, early (<14 days of onset of ARDS) and late phases (after 14 days of onset of ARDS).

### Corticosteroids in early ARDS

Early ARDS is characterized by a strong proinflammatory response and one would expect that antiinflammatory drugs must be beneficial at this stage. However, trials with therapeutic corticosteroids in established cases of early ARDS showed variable results.[21–25] Initially, Bernard *et al*. tried short course of high-dose MP (30 mg/kg body weight every six hours for 24 hours) in 50 patients with ARDS.[21] This study demonstrated insignificant beneficial effects of MP, such as reduced 45-day mortality (60% vs 63%) and increased chance of reversal of ARDS (39% vs 36%) as compared with placebo (*P* = 0.07).[21] All subsequent trials are with prolonged therapeutic corticosteroids which showed variable results [Table 2]. In all these studies, mortality (at various intervals during therapy) was taken as main outcome to document benefit of the therapy. Other clinically important parameters which have been studied were organ dysfunction score, lung injury score, oxygenation, duration of mechanical ventilation, and duration of ICU stay which have significant impact on cost of treatment.

**Table 2:** Trials of corticosteroids for treatment of ARDS

Author, Year^[Ref]^	Drug/dosage	No. death/total no. patients with ARDS (%)		OR (95% CI)	*P* value
		Corticosteroid group	Placebo group		
Bernard *et al*. 1987[21]	Methylprednisolone 30 mg/kg IV 6 hourly for 24 hours.	30/50 (60)	31/49 (63.2)	0.75 (0.4-1.57)	0.74
Meduri *et al*. 1998[22]	Protocol-based IV methylprednisolone^a^.	2/16 (12.5)	5/8 (62.5)	0.41 (0.06-99)	0.03
Annane *et al*. 2006[23]	Hydrocortisone 50 mg IV 6 hourly and 9-alpha fludrocortisone once a day for 7 days.	33/62 (53)	50/67 (75)	0.35 (0.15-0.82)	0.16
Steinberg *et al*. 2006[24]	Protocol-based IV methylprednisolone^b^.	26/89 (29.2)	26/91 (28.5)	0.84 (0.40-1.60)	1.00
Meduri *et al*. 2007[25]	Protocol-based IV methylprednisolone^c^.	15/63 (23.8)	12/28 (42.8)	0.53 (0.21-1.21)	0.03

ARDS: Acute respiratory distress syndrome

a Loading dose of 2 mg/kg; then 2 mg/kg/d from day 1 to day 14, 1 mg/kg/d from day 15 to day 21, 0.5 mg/kg/d from day 22 to day 28, 0.25 mg/kg/d on days 29 and 30, and 0.125 mg/kg/d on days 31 and 32. In patients who were extubated prior to day14, treatment was advanced to day 15 of drug therapy and tapered according to schedule

b Loading dose of 2 mg/kg of predicted body weight followed by 0.5 mg/kg 6 hourly for 14 days; 0.5 mg/kg 12 hourly for 7 days; and then tapering of the dose

c Loading dose of 1 mg/kg followed by an infusion of 1 mg/kg/d from day 1 to day 14, 0.5 mg/kg/d from day 15 to day 21, 0.25 mg/kg/d from day 22 to day 25, and 0.125 mg/kg/d from day 26 to day 28. In patients who were extubated between days 1 and 14 were advanced to day 15 of drug therapy and tapered according to schedule

Mortality data from various trials showed variable results [Table 2]. A large trial by ARDS clinical trial network (ARDSnet), consisting of 180 patients with ARDS (both early [73%] and late [27%]), showed that MP (2 mg/kg bolus in first 24 hours, followed by a dose of 0.5 mg/kg every 6 hours for 14 days, 0.5 mg/kg every 12 hours for 7 days, and then tapering over 4 days) has no survival benefits. It showed that in patients with early ARDS (enrolled 7-13 days after onset of ARDS), there were no significant differences in 60-day mortality (36% *vs* 27%; *P* = 0.26) in the placebo and in MP group.[24] Similar results were seen at 180-day with mortality of 31.9 and 31.5% in the placebo and MP group, respectively. Other workers have also shown that corticosteroids use have no statistically significant mortality benefits in patients with ARDS.[23,24]

Meduri *et al*., however, showed that prolonged corticosteroids use (a loading dose of 1 mg/kg, followed by an infusion of 1 mg/kg/day from day 1 to day 14, 0.5 mg/kg/day from day 15 to day 21, 0.25 mg/kg/day from day 22 to day 25, and 0.125 mg/kg/day from day 26 to day 28) in early ARDS was associated with significantly decreased ICU mortality (20.6% *vs* 42.9%; *P* = 0.03).[25] Of note, patients were enrolled within 72 hours of entry to the study, thereby insuring corticosteroids early in the course of disease. This study may be criticized for poor matching between two groups as incidence of catecholamine-dependent shock in placebo group was nearly twice as compared with corticosteroids group (46.4% *vs* 23.8%). There is enough evidence that vasopressor use is an independent predictor of mortality and might have contributed to increased mortality in placebo group.[40,41] Second limitation of this study is that a significant percentage of control patients crossed over to receive open-label MP; however, data were analyzed as intention to treat analysis. Intention to treat analysis is meant for larger trials but when same is applied to the smaller trials, the results may be biased by protocol violation and per protocol analysis is the best method.

These conflicting conclusions may be a result of differences in characteristics of study cohort and treatment protocol of these studies. In ARDSnet trial, there was poor matching for age, gender, pneumonia, trauma, serum creatinine, APACHE III, compliance, and lung injury score. All these factors can affect the final outcome. Furthermore, in ARDSnet trial,[24] there was rapid tapering of corticosteroids, as compare with cohort studied by Meduri *et al*.,[25] which might have contributed to increased mortality in this trial. Another important difference between these two trials is the timing of administration of corticosteroids. In ARDSnet trial corticosteroids were used during unresolving or persistent ARDS (less than 14 day). However, pathological ARDS has been classified as “early” (from 1-7 days after onset), “early persistent” (days 7-14), and “late persistent” (>14 days).[42] Conceptually, corticosteroids administration should be more effective during exudative phase (early phase). Unfortunately, clinically, it is difficult to identify different phases of ARDS without lung biopsy; however, its role in clinical practice is yet to be defined. The pooled data from recently conducted large trials[23–25] showed that patients who received corticosteroids early (<14 days after onset of ARDS) had reduced mortality (82/214 [38%] vs 98/186 [52.5%], relative risk (RR) 0.78; 95% CI [0.64–0.96]; *P* = 0.02).[43]

There are many clinical parameters which are important contributors to the morbidity associated with ARDS, such as organ dysfunction score, lung injury score, oxygenation, duration of mechanical ventilation, and ICU stay, as these have significant effect on the cost of treatment. Therefore, the effect of corticosteroids on these parameters would be important consideration for its use in ARDS. ARDSnet trial showed that MP increased the number of ventilator-free days (14.1 days *vs* 23.6 days, *P* = 0.006). Similar results were shown in other trials.[23,25] Length of ICU stay is an important factor which uses lots of resources. Meduri *et al*. showed that corticosteroids use leads to significant reduction in number days of ICU stay (7 *vs* 14.5 days; *P* = 0.007).[25] In ARDSnet trial also, ICU stay was significantly lower in patients who received corticosteroids.[24] Similar findings has been reported by various other studies.[43] The corticosteroids have been shown to reduce disease severity scores, namely, the multiple organ dysfunction syndrome score and lung injury score. These agents also improve oxygenation (PaO2/FIO2 ratios).[44]

### Corticosteroids in late ARDS

Persistent ARDS at later stages is characterized by more of fibrosis than cellular inflammation; therefore, corticosteroid effect is expected to be different. It has been observed that there is significant association between late initiation corticosteroids and failure to improve, with 50% failure rate (*P* = 0.04).[22] In ARDSnet trial in patients with late ARDS (enrolled 13 days after onset of ARDS), corticosteroids use was associated with increased mortality (35% vs 8%; *P* = 0.02) and neuromuscular weakness.[24] However, it should be noted that the mortality in the control arm is unexpectedly low and one may question whether this group is true representation of late ARDS. Pooled data of trials done over the last more than 20 years, which include patients with both early and late stage of ARDS, did not show significant mortality benefits (Odd’s Ratio, 0.62 with 95% CI of 0.23-1.26).[45] However, data of only recent trials which consist predominantly of early ARDS patients showed beneficial effect on clinically significant parameters.[43] These results suggest that corticosteroid use in late stage of ARDS probably have negative effect on final outcome.

## ADVERSE EFFECTS OF CORTICOSTEROIDS

Corticosteroids are potential therapeutic agents which are used in critically ill patients for various indications including ARDS. However, these agents have number of adverse effects, such as hyperglycemia, fluid retention, electrolyte imbalance, pancreatitis, increased infection rate, neuromuscular weakness, gastrointestinal (GI) bleed, etc, which may be important limiting factor of this therapy. Adverse events related to corticosteroids use in ARDS have been reported variably by the different authors.[22–25] ARDSnet trial showed that rate of new infections was lower in patients receiving MP (22.4%) as compared with placebo (32.9%), though results were not statistically significant (*P* = 0.14).[24] Meduri *et al*. reported similar infection rate in both the groups and no GI bleed.[22] Study by Annane *et al*. showed that rates of superinfection (13% *vs* 12%), GI bleed (2% vs 6%), and psychiatric disorders were similar in both placebo and corticosteroids groups.[23] Similarly, Meduri *et al*. showed that corticosteroids were safe to use in patients with ARDS, where 27/63 (42.9%) in MP group and 17/28 (60.7%) in placebo group developed new infection.[25] Hyperglycemia was observed in ARDSnet trial with MP group having significantly higher glucose. However, Meduri *et al*. have shown that patients requiring insulin to treat hyperglycemia were similar (71.4% *vs* 64.3%) with MP and placebo.[25] ARDSnet trial reported similar incidence of neuromuscular weakness in both placebo and treatment arm (24% *vs* 29%). However, serious neuromuscular weakness was observed in 9 patients, all were in MP group (*P* = 0.001).[24] However, study by Meduri *et al*. showed similar incidence of neuromuscular weakness (6.4% *vs* 3.6%) in patients treated with MP as compared with placebo.[25] Other side effects observed were pancreatitis and GI bleed.[25] A recent meta-analysis has shown that there was no difference in the incidence of infection, neuromyopathy, GI bleeding, and life-threatening complications, such as major organ failure (heart, kidney, and liver), between corticosteroid and placebo group.[44]

It can be concluded that corticosteroids are relatively safe drugs to use in patients with ARDS. However, a close surveillance, as described by Meduri *et al*. and ARDSnet trial, is recommended to identify the new infection and treat it promptly, as significant number of patients on corticosteroids may develop infection in the absence of fever. Along with this, appropriate glycemic control and minimal use of sedation and neuromuscular blockade will further minimize the complication rate.

## PROBABLE REASONS FOR CONTROVERSIAL RESULTS

These controversies may be results of analyses based on a small number of trials with sparse data in treatment arm which might have limited power for detection of important outcome like mortality. This is further complicated by stratification of studies into two subgroups: preventive and therapeutic. Other significant problem with these trials is the fact that it is almost impossible to standardize all parameters in critically ill patients. This results in significant heterogeneity in study cohort even after the best efforts at randomization. Various factors which may be responsible for this heterogeneity are as follows: etiology of ARDS (pulmonary *vs* extrapulmonary), respiratory mechanics, inflammatory response, and ventilator strategies used to treat different patients. It is not possible to standardize all these parameters and it is likely that patients in different trials may not be comparable. However, all these factors may significantly alter the final outcome. Therefore, it should not be surprising to find variable results ranging from significant benefit to no benefit of corticosteroids in heterogeneous population of ARDS.

## CONCLUSION

Despite sound physiological basis, corticosteroids have not shown clear cut benefit in management of ARDS. It is clear from the available data that these agents have no role in prevention and late phase of ARDS. However, there is silver lining in the management of early ARDS using these agents. There is distinct advantage of corticosteroids in improving organ function score, lung injury score, and oxygenation which result in reduction in duration of mechanical ventilation requirement and ICU stay, although only one study showed mortality benefits. Heterogeneity of the conditions causing ARDS may be the reason for the lack of uniformity in the results.

More studies are required to have convincing data regarding corticosteroid use in early ARDS before any definitive treatment recommendation. Future trials with corticosteroids may be designed in such way to make this cohort as homogenous as possible, so that we can identify the patients who will be benefited with such therapy.
